# Long-Term Follow-Up of Disability, Cognitive, and Emotional Impairments after Severe Traumatic Brain Injury

**DOI:** 10.1155/2019/9216931

**Published:** 2019-08-27

**Authors:** Britt-Marie Stålnacke, Britt-Inger Saveman, Maud Stenberg

**Affiliations:** ^1^Department of Community Medicine and Rehabilitation, Rehabilitation Medicine, Umeå University, Umeå, Sweden; ^2^Department of Nursing, Umeå University, Umeå, Sweden

## Abstract

**Aim:**

To assess the clinical course of disability, cognitive, and emotional impairments in patients with severe TBI (s-TBI) from 3 months to up to 7 years post trauma.

**Methods:**

A prospective cohort study of s-TBI in northern Sweden was conducted. Patients aged 18-65 years with acute Glasgow Coma Scale 3-8 were assessed with the Glasgow Outcome Scale Extended (GOSE), the Hospital Anxiety and Depression Scale (HADS), and the Barrow Neurological Institute Screen for Higher Cerebral Functions (BNIS) at 3 months, 1 year, and 7 years after the injury.

**Results:**

The scores on both GOSE and BNIS improved significantly from 3 months (GOSE mean: 4.4 ± 2.3, BNIS mean: 31.5 ± 7.0) to 1 year (GOSE mean: 5.5 ± 2.7, *p* = 0.003, BNIS mean: 33.2 ± 6.3, *p* = 0.04), but no significant improvement was found from 1 year to 7 years (GOSE mean: 4.7 ± 2.8, *p* = 0.13, BNIS mean: 33.5 ± 3.9, *p* = 0.424) after the injury. The BNIS subscale “speech/language” at 1 year was significantly associated with favourable outcomes on the GOSE at 7 years (OR = 2.115, CI: 1.004-4.456, *p* = 0.049).

**Conclusions:**

These findings indicate that disability and cognition seem to improve over time after s-TBI and appear to be relatively stable from 1 year to 7 years. Since cognitive function on some of the BNIS subscales was associated with outcome on the GOSE, these results indicate that both screening and follow-up of cognitive function could be of importance for the rehabilitation of persons with s-TBI.

## 1. Introduction

Traumatic brain injury (TBI) is a major public health problem all over the world and is a leading cause of death and long-term disability in people of working age [1]. The most widely used TBI classification is based on the level of consciousness (LOC) on admission, defined by the Glasgow Coma Scale (GCS) [2]. The annual incidence of all levels of traumatic brain injury (TBI; mild, moderate, and severe according to GCS 3-15) is 250–350,000/year [3–5]. Severe traumatic brain injury (s-TBI) varies between GCS of 3 and 8 on admission; is much rarer, with incidence estimates of 3–12/100,000/year [4]; and has the highest mortality rate [6]. The damage constitutes a major health problem and causes great suffering for both patients and their families with extensive consequences both at the individual and the community level. Patients with s-TBI comprise a heterogeneous group with deficits and impairments of varying complexity that can affect both the physical and mental status as well as social and family life [7]. Cognitive deficits and emotional impairments including symptoms of depression and anxiety have been shown to affect the achievement of rehabilitation goals after TBI and are a major cause of disability after TBI [8]. Since cognitive and emotional impairments are not noticeable initially, it is difficult for other people to understand the severity and problems a person with s-TBI is having to cope with.

Various scales have been developed for the assessment of cognition, depression, anxiety, and disability after TBI. Most studies of cognitive deficits have focused on deficits in memory, attention/concentration, and visuospatial abilities [9, 10], but the impact on a combination of these areas together with affective functions, orientation, and awareness has not been studied to the same extent. The Barrow Neurological Institute Screen for Higher Cerebral Functions (BNIS) is an instrument that has been used to measure both cognitive and affective disturbances in patients with TBI [11]. The prevalence of depression and anxiety after TBI has been shown to vary widely and may be related to different methods of assessment. One of the most common instruments used in clinical healthcare is the Hospital Anxiety and Depression Scale (HADS) [12] that has been studied in a large number of different patient groups. To measure outcome after s-TBI, the Glasgow Outcome Scale (GOS) is commonly used [13, 14]. This is a global and somewhat crude instrument that only roughly discriminates different levels of disability. A more sophisticated version of the GOS, the Glasgow Outcome Scale Extended (GOSE), has thus been developed, allowing for a more fine-tuned categorisation of posttraumatic disability.

Most studies of s-TBI focus on the acute phase and then a short follow-up period. In a previous national multicentre study of patients with s-TBI (the ProBrain study), we assessed the clinical course of cognitive and emotional function from 3 weeks to 1 year after trauma [15].

In the present study, the aim was to assess the clinical course of patients with s-TBI from northern Sweden (a subgroup of the ProBrain population) on disability, cognitive, and emotional impairments up to 7 years after trauma.

## 2. Methods

### 2.1. Design

The design of the study is a prospective cohort study.

### 2.2. Patients

Inclusion criteria for the study were patients aged 18–65 years, with acute s-TBI with the lowest nonsedated GCS (Glasgow Coma Scale) [2] score 8 of 3–8 within 24 hours post trauma. The exclusion criterion for this study was death within 3 weeks of injury. Thirty-seven patients (11 female, 26 male) with s-TBI were admitted from January 2010 to December 2011 to the Neurotrauma Center (NC) at Umeå University Hospital. These 37 patients were included as part of the Swedish-Icelandic multicentre ProBrain study, a prospective 2-year study with a one-year follow-up of severe traumatic brain injuries [15].

### 2.3. Procedure

The 37 patients with s-TBI were treated in the acute phase at the Neurotrauma Center (NC) at Umeå University Hospital, covering the North Health Region (NHR) which covers a geographical area which comprises almost half of the total area of Sweden (136,373 km^2^). It is divided into four counties and has approximately 900,000 inhabitants, thus comprising only 10% of the total national population. Patients were evaluated at 3 months, 1 year, and up to 7 years after the injury. Assessment took place in the patients' current care setting or at their homes and was performed by the same rehabilitation physician at all time points.

### 2.4. Instruments

#### 2.4.1. The Barrow Neurological Institute Screen for Higher Cerebral Functions (BNIS)

The Barrow Neurological Institute Screen for Higher Cerebral Functions (BNIS) [11] is a cognitive screening test for speech and language functions, orientation, attention/concentration, visuospatial and visual problem solving, memory, affect, and awareness. BNIS yields a total score and seven subscale scores. The total score (maximum 50 points) reflects overall cognitive function and consists of the results from a prescreen plus the 7 subscale scores (speech and language, 15 points; orientation, 3 points; attention/concentration, 3 points; visual and visuospatial problem solving, 8 points; memory, 7 points; affect, 4 points; and awareness, 1 point). A total subscale score can be obtained. Higher scores reflect a higher level of functioning. If the total BNIS score is below 47 points, further cognitive investigation is recommended. The BNIS has been validated for a Swedish population [16, 17].

#### 2.4.2. The Hospital Anxiety and Depression Scale (HADS)

The Hospital Anxiety and Depression Scale (HADS) [12] was used to identify the presence and degree of anxiety and depression. It consists of 14 items (7 items in each subscale, HADS-depression (HADS-D) and HADS-anxiety (HADS-A)) which are assessed on a 4-point Likert scale (range 0-3), where the total score is the sum of each subscale (range 0-21). Cut-offs for both subscales of 8 or higher were used to determine “caseness” [12]. The HADS is an established screening tool for anxiety and depression and it has been used previously in patients with TBI. The HADS has acceptable reliability, sensitivity and specificity in assessing symptom severity in anxiety and depression in various populations [18].

#### 2.4.3. The Glasgow Outcome Scale Extended (GOSE)

The Glasgow Outcome Scale Extended (GOSE) [19] extends the 5 categories of the previously developed Glasgow Outcome Scale (GOS) [19] to 8 on disability, thereby increasing its sensitivity. The 8 categories span from “death” (score 1) to “upper good recovery” (score 8). GOSE was also dichotomised into “unfavourable outcome” (GOSE 1-4), and “favourable outcome” (GOSE 5-8). The GOSE has good interrater reliability [19] and validity [20] and is an established measure of global outcome after traumatic brain injury.

## 3. Statistical Analysis

Data were analysed with SPSS, version 25, for Windows. Data were reported as frequencies or median and means. Nonparametric tests were used as the samples were small and/or not normally distributed. Thus, Wilcoxon's signed-rank test was used for the study of paired observation variables. The level of statistical significance was set as *p* = 0.05. Univariate binary logistic regression analyses were performed to explore associations between the following variables at 1 year: BNIS (total score and the 7 subscale scores), HADS-A, and HADS-D with GOSE as a dependent variable at follow-up after 7 years (“unfavourable and favourable outcomes”). Variables that had a *p* value < 0.1 in the univariate regression analyses were then included in a multivariate model using a forward method. The results of the logistic regression analyses are presented as an odds ratio (OR). The reliability of the OR is expressed as a 95% confidence interval (CI). Statistical significance was set at *p* < 0.05 for the multivariate regression analysis.

## 4. Ethical Considerations

The study has been approved by the Research Ethical Review Board Umeå Sweden, D-nr. 2016/444-31.

## 5. Results

At the time of follow-up, 9 participants were deceased. Six of them (4 men and 2 women) had died within the first year and three later on. GCS 3 was seen in 9 of the included patients and hospital deaths occurred in 4 of these patients. Two of these patients died at the NC due to respiratory complications, and traumatic and inoperable intracranial aneurysm, respectively. One of the fatalities suffered from multiple illnesses at the time of injury, two of the patients with very severe brain injury (GCS 3) died because of respiratory infection/infection/pneumonia complications, and one patient died from intracerebral bleeding after transportation from NC to the local hospital. Patients with hospital death had a significantly higher mean age in comparison with patients who survived (52.8 ± 17.8 vs. 41.3 ± 15.1, *p* = 0.048).

Thus, there were 28 remaining patients with s-TBI who were eligible to participate in this study. Three participants could not be identified, and four patients declined to participate. Therefore, 21 persons were included in the 7 years follow-up (14 men and 7 women). They were aged between 17 and 64 years at the time of the injury (mean 41 years). Their age varied from 27 to 70 years (median 49) at the time of the follow-up. The median for the lowest nonsedated GCS within 24 hours post trauma was GCS 5 (3-8). Causes of the injury were falls in 9 cases and traffic incidents in 8 cases (Table 1). The participants with s-TBI were hospitalised on average 62 (16-250) days and in a specialised rehabilitation department for an average of 35 (0-127) days.

For background information, see Tables 1 and 2. The number of part-time/full-time workers or being a student had decreased from 17 at time for injury to 10 after 7 years, and participants on full or partial sick leave had increased from 3 to 9. Four participants had personal assistants 24/7 or social care several times daily at follow-up. The ability to keep or get a driving license had decreased from 16 to 13 of the injured participants (Table 2). Eight of the participants with s-TBI had returned to earlier employment or retired. One of the participants with s-TBI also had an incomplete traumatic spinal cord injury but could walk at follow-up.

### 5.1. GOSE

GOSE improved significantly from 3 months (median 4.5 (1-8), mean: 4.4 ± 2.3) to 1 year (median 7 (1-8), mean: 5.5 ± 2.7; *p* = 0.003) and close to significantly from 3 months and 7 years (median 5 (1-8), mean: 4.7 ± 2.8; *p* = 0.059). No significant improvement was found on GOSE from 1 year to 7 years (*p* = 0.137). At 3 months, GOSE 1-4 was seen in 50% and GOSE 5-8 in 50%. At 1 year, GOSE 1-4 was seen in 36% and GOSE 5-8 in 64%. At 7 years, GOSE 1-4 was seen in 42% and GOSE 5-8 in 58%. Good recovery (GOSE 7-8) was seen in 59%, both at 1 year and at 7 years follow-up (see Table 3).

### 5.2. Hospital Anxiety and Depression Scale-Depression (HADS-D) Scores

No statistically significant differences were found for HADS-D from 3 months (median: 2 (0-10), mean: 3.1 ± 3.0) to 1 year (median: 2.5 (0-12), mean: 3.4 ± 2.5; *p* = 0.793), from 3 months to 7 years (median: 3 (0-11), mean: 3.6 ± 3.0; *p* = 0.875), and from 1 year to 7 years (*p* = 0.391). HADS-D scores above cut-off occurred in 3 participants at 3 months, in 5 participants at 1 year, and in 3 participants at 7 years after injury (see Table 3).

### 5.3. Hospital Anxiety and Depression Scale-Anxiety (HADS-A) Scores

There was a close to significant difference between the scores of HADS-A from 3 months (median: 3.5 (0-14), mean: 4.5 ± 3.8) to 1 year (median: 4 (0-12), mean: 3.8 ± 3.5; *p* = 0.059), but not from 3 months to 7 years (median: 3 (0-13), mean: 3.7 ± 3.7; *p* = 0.373) and not from 1 year to 7 years (*p* = 0.699). HADS-A scores above cut-off occurred in 5 participants at 3 months, in 4 participants at 1 year, and in 3 participants at 7 years after injury (see Table 3).

### 5.4. BNIS

The BNIS total subscale score improved significantly on the paired *t*-tests from 3 months to 1 year post injury (*p* = 0.042). From 1 year to 7 years, no further improvement was found.

No significant improvement on the separate BNIS subscales was shown from 3 months to 7 years after the injury (see Table 4).

### 5.5. Outcome as Assessed with the Glasgow Outcome Scale Extended (GOSE)

In the univariate logistic regression analysis GOSE after 7 years as a dependent variable, several variables 1 year after the injury had a *p* value < 0.1. Associations (*p* value < 0.1) were found between the following variables after 1 year and “favourable outcome” on the GOSE: BNIS total subscale score (OR = 1.197, CI: 0.985-1.455, and *p* = 0.070), BNIS subscale speech/language (OR = 2.115, CI: 1.004-4.456, and *p* = 0.049), visuospatial and visual problem solving (OR = 1.765, CI: 0.964-3.232, and *p* = 0.066), and memory (OR = 1.772, CI: 0.933-3.365, and *p* = 0.080). No associations were found between the BNIS subscales orientation, affect, awareness, HADS-A, and HADS-D after 1 year and GOSE at 7 years. The three BNIS subscales speech/language, visuospatial and visual problem solving, and memory were incorporated into a multivariate model. The analysis showed that statistically significant associations were only obtained for speech and language (OR = 2.115, CI: 1004-4.456, and *p* = 0.049).

## 6. Discussion

This study shows that both disability measured with the GOSE and cognition with the BNIS improved significantly from 3 months to 1 year while no further improvement was shown 7 years after the injury.

Nine of the included patients were deceased at the time of follow-up. The majority (six) had died within the first year and three later on. In the group of patients that could be followed up 7 years after the severe traumatic injury, the level of recovery and function varied hugely, from two of the participants having personal assistants all day and night to ten who were working or studying part- or full time. However, the patients who died were older and had lower GCS compared with the survivors. As was the case in previous studies, the majority were males [3, 21]. The severity of TBI on the acute GCS (median GCS) was consistent with other prospective studies of s-TBI [20]. Falls are a common cause of TBI, and in some previous Scandinavian studies, falls have been reported to cause most incidents of s-TBI [3, 21]. Falls were also the most frequent cause of injury in our population of working aged adults. The second most common cause of injury in this study was transport accidents which in American and Australian studies is reported as being the predominant cause [22]. Indications of alcohol use at the time of injury have been shown to be a risk factor for TBI [23, 24]. In our study, almost half were under the influence of alcohol and/or drugs at the time of injury. This is a much higher rate of alcohol use in patients with s-TBI than that reported in a Norwegian study (32%) [25] and in previous studies of patients with m-TBI conducted at the same university hospital as the present study [26]. These findings indicate the existence of high-risk populations and the need for preventive measures.

s-TBI is a heterogeneous group with various deficits and long-term outcomes. This was also shown in our population. Although the patients were initially classified as severe TBI with low GCS scores [2], 1 year after the injury, the overall outcome was encouragingly favourable (GOSE 5-8, 65%) with significantly increased scores on the GOSE. However, this positive trend did not continue over the coming years on the GOSE as the scores had decreased at 7 years. Similar results were found regarding the BNIS at 1 year with higher total scores compared with 3 months but no further improvement. These findings of GOSE recovery plateauing 1 year after the injury are in accordance with the results reported in another study [27] of patients with moderate and severe TBI examined from 3 months to 24 months after the injury. The lower GOSE scores at 7 years could be seen in the concept of TBI as a chronic health condition where factors such as age and preinjury employment may play a role for the long-term outcome [28].

There are probably several reasons for the varying long-term results in the present study. In a previous study of the same population of patients with s-TBI, we studied the clinical pathways 3 months after the severe traumatic injury in the rural area of the North Health Region that covers almost half of Sweden. Most patients were swiftly transported direct to the regional well-monitored neurosurgical and neurointensive care unit. In contrast, the postacute clinical pathways less clearly reflected an optimised medical and rehabilitative strategy. Patients were often transferred back to local hospitals at a fairly early stage [29]. Since subsequent treatment and rehabilitation interventions varied after injury, there were probably no further long-term follow-up strategies from the healthcare services for these patients.

As s-TBI comprises injuries with wide variations as regards complexity of impairments and functional outcomes, prognostic indicators are essential for rehabilitation and treatment.

In a study of prognostic factors for s-TBI after 1 year of the same study population, we used two classification systems of computed tomography (CT) of the brain in the acute phase for prognostication and a prognostic calculator protocol (Corticosteroid Randomisation after Significant Head Injury (CRASH) acute calculator protocol). Neither CT nor CRASH yielded clinically useful predictions of outcome at 1 year post injury [30]. In the present study, the BNIS subscales speech/language, visuospatial and visual problem solving, and memory at 1 year were significantly or close to significantly associated with “favourable outcome” on the GOSE at follow-up after 7 years. However, in the multivariate model, significant association was only obtained for speech and language. This result is in line with the previous multicentre study ProBrain which reported relationships between some of the BNIS subscales and outcome on the GOSE 1 year after the injury [15].

Although the BNIS scores improved over time in our study, the scores at 1 and 7 years were in a range that was similar to that reported by Swedish TBI patients from a neurorehabilitation clinic [17] which could indicate that the long-term results are probably relatively consistent. However, it is worth remembering that the BNIS is a screening instrument that can be used to detect patients in need of comprehensive cognitive neuropsychological assessment.

This study has several strengths, such as a prospective design and close collaboration as regards case identification, which meant it was possible for most patients to be included. In addition, the extant protocols allowed for referral of patients with a seemingly dismal prognosis. Furthermore, one of the authors (MS) examined all patients at 3 months, at 1 year, and at 7 years and ensured that data were precisely and completely documented, making the amount of missing or secondary data minimal. However, the study is limited by the relatively small study population, of s-TBI, but the subset of this severe group of TBI is rare in comparison with the great majority of mild-moderate TBI. Nevertheless, the included patients comprised a near-total regional cohort population of incident s-TBI patients injured over a period of two years. Most prior studies using the BNIS have used heterogeneous study populations with a mix of diagnoses and different TBI grades [17, 31] which were studied at different time points after the trauma.

A limitation of the study is that not all patients could be followed up, but only three participants could not be identified and another four declined to participate. Another limitation is the heterogeneity of the age of the participants. At follow-up 7 years after the injury, the youngest patient was 27 years old and the oldest was 70 years old. Since outcomes may differ in different age groups, this could be seen as a considerable methodological limitation. However, the study population could reflect the real-life population of persons with s-TBI initially treated at a university hospital.

## 7. Conclusion

The results indicate that cognition on the BNIS improves over time after s-TBI and appears to be relatively stable from 3 months to 1 year. Since no further improvement was seen at 7 years, the long-term results are probably relatively consistent. Cognitive function on some of the BNIS subscales was associated with outcome on the GOSE. Therefore, it seems that both screening and follow-up of cognitive function could be of importance for the rehabilitation of persons with s-TBI.

## Figures and Tables

**Table 1:** Demographic injury characteristics for persons with severe traumatic brain injury in northern Sweden (approximately 900,000 inhabitants).

	*N*	%
Included year: 2010-2011	37	100
Included at follow-up	21	57
Age at follow-up (*n* = 21)		
Median 49 (27-70) years		
Gender (*n* = 21)		
Male	14	66
Female	7	33
Diagnosis (*n* = 21)		
S062 diffuse brain injury	6	29
S063 focal brain injury	2	10
S064 EPH	4	19
S065 traumatic SDH	7	33
S066 traumatic SAH	1	5
S068 other	1	5
Influence of alcohol misuse at time of injury	18	
Cause of injury (*n* = 21)	9	43
Fall	8	38
Traffic	3	14
Sport	1	5
Unknown		
Education (*n* = 21)	10	48
<12 years	8	38
12 years	3	14
>12 year		

EPH: epidural hematoma; SDH: subdural hematoma; SAH: subarachnoidal hematoma.

**Table 2:** Marital status, employment and livelihood, personal support, driving license at time of injury (2010-2011) and at follow-up after 7 years (*n* = 21).

	Time of injury		Follow-up after 7 years	
	*n*	%	*n*	%
*Marital status*				
Unmarried without underage children living at home	4	19	4	19
Unmarried with underage children living at home	1	5	0	0
Married/cohabitation without underage children living at home	9	43	10	48
Married/cohabitation with underage children living at home	6	29	5	24
Married/cohabitation with grown child living at home	1	5	1	5
*Employment*				
Working or studying part time or full time	17	81	10	48
Sick leave full time or part time	3	14	9	43
Social care	1	5	1	5
Other	0	0	1	5
*Personal support*				
Personal assistant 24/7	0	0	2	10
Social care several times daily	0	0	2	10
*Driving license*	16	76	13	62

**Table 3:** HADS-depression (HADS-D), HADS-anxiety (HADS-A), and GOSE scores at 3 months, 1 year, and 7 years.

	3 months	1 year	3 months versus 1 year (*p* value)	7 years	3 months versus 7 years (*p* value)	1 year versus 7 years (*p* value)
HADS-D total score median (range)	2 (0-10)	2.5 (0-12)	0.793	3.0 (0-11)	0.875	0.391
HADS-D mean (SD)	3.1 (3.0)	3.4 (2.5)		3.6 (3.0)		
HADS-A total score median (range)	3.5 (0-14)	4 (0-12)	0.059	3.0 (0-13)	0.373	0.699
HADS-A mean (SD)	4.5 (3.8)	3.8 (3.5)		3.7 (3.7)		
*HADS* − *D* ≥ 8% (*n*)	12.0 (3)	17.9 (5)	0.694	8.1 (3)		0.442
*HADS* − *A* ≥ 8% (*n*)	19.2 (5)	14.3 (4)	0.085	15.0 (3)		0.216
GOSE median (range)	4.5 (1-8)	7 (1-8)	0.003	5 (1-8)	0.059	0.137
GOSE mean (SD)	4.4 (2.3)	5.5 (2.7)		4.7 (2.8)		
GOSE 1-4 (*n* %)	48.6	35.1	0.001	42		0.001
GOSE 5-8 (*n* %)	48.6	64.9		58		

**Table 4:** BNIS subscale scores and total BNIS scores at 3 months, 1 year, and 7 years.

BNIS score	3 months (*n* = 25) median (range)	1 year (*n* = 28) median (range)	3 months vs. 1 year (*p* value)	7 years (*n* = 13) median (range)	1 year vs. 7 years (*p* value)
Speech/language	14 (10-15)	14 (10-15)	0.398	15 (9-15)	0.713
Orientation	3 (1-3)	3 (2-3)	0.317	3 (2-3)	1.000
Attention/concentration	2 (0-3)	2 (1-3)	0.392	2 (1-3)	0.317
Visuospatial and visual problem solving	6 (2-8)	6 (1-8)	0.054	6 (3-5)	0.496
Memory	4 (1-7)	5 (0-7)	0.143	4 (1-6)	0.226
Affect	4 (2-4)	3 (2-4)	0.477	4 (2-4)	0.739
Awareness	1(0-1)	1 (0-1)	0.705	0 (0-1)	0.257
Total BNIS subscale score mean (SD)	31.5 (7.0)	33.2 (6.3)	0.042	33.5 (3.9)	0.424

## Data Availability

The datasets generated and/or analysed in this study are not publicly available as the Ethical Review Board has not approved the public availability of these data.
